# Mediastinal Germ-cell Tumors Relapse in a Male With Klinefelter Syndrome. Is Longer Surveillance Needed?

**DOI:** 10.1097/MPH.0000000000002837

**Published:** 2024-03-06

**Authors:** Francesca Stefanachi, Maria Carmen Affinita, Giulia Fichera, Arianna Tagarelli, Federica De Corti, Federico Rea, Gianni Bisogno

**Affiliations:** * Department of Woman’s and Children’s Health, Hematology and Oncology Unit; † Pediatric Radiology Unit, University Hospital of Padova; ‡ Department for the Health of Woman and Child, Pediatric Surgery Division; § Thoracic Surgery Unit, Department of Cardiologic, Thoracic and Vascular Sciences, University Hospital of Padova, Padova, Italy

**Keywords:** Klinefelter syndrome, germ-cell tumor, teratoma, children

## Abstract

Germ cell tumors (GCTs) are a heterogeneous group of pediatric cancers. In up to one-third of male patients, a primary mediastinal location is associated with the presence of Klinefelter syndrome (KS). We describe a case of mediastinal GCT in a patient, with unacknowledged KS, that presented a relapse 7 years from diagnosis, that is, 2 years after the end of the follow-up program usually recommended for patients with GCT. There are no recommendations for screening for KS in patients with mediastinal GCT and there are no specific guidelines for surveillance of GCT in KS patients. Our experience suggests that KS should be suspected in patients with mediastinal GCT, and a longer follow-up plan should be implemented when GCT occurs in patients with KS.

Germ cell tumors (GCTs) represent 3% of pediatric cancers and more than 10% of tumors in adolescents.^1^ In the pediatric population, more than half of GCT occurs in the extra-gonadal site (eGCT), more commonly in the sacrococcygeal region in younger children and in the central nervous system during and after puberty.^2,3^

With modern multidisciplinary approaches, the GCT overall survival is above 90%. Age of older than 11 years, extra-gonadal site, and the presence of metastasis are described as poor prognostic factors.^1^ Most patients achieve tumor complete remission by the end of treatment. The first 2 years from diagnosis are the period at higher risk of relapse^4^ but late events are described and a 5 years follow-up is recommended in all patients.^5^

GCT may be associated with genetic disorders including Klinefelter syndrome (KS), Turner syndrome, Swyer syndrome, and others.^1^ Patients with KS present an increased risk of eGCT in comparison to the general population.^6^ In these patients, the mediastinum is the most frequent primary location and teratoma is the most frequent histology.^7^

We describe a late relapse of a mediastinal teratoma that occurred in a patient with KS 7 years after the first complete remission, to discuss the follow-up program in syndromic patients.

Patient-informed and written consent was obtained for this report. Authors have no conflicts of interest to declare.

## CASE REPORT

An 11-year-old male patient presented with chest pain, fatigue, and fevers. Chest radiograph and CT scan revealed an expansive 11×9×15 cm mediastinal mass occupying the left hemithorax with radiologic features consistent with a teratoma.

Alpha-fetoprotein was moderately increased (49 ng/dL) and β-human chorionic gonadotropin was normal.

The patient underwent surgery, which resulted in microscopically complete resection of the lesion. Histologic analysis confirmed a mixed GCT tumor with 95% mature teratoma and a small and isolated component of immature neuroectodermal tissue, with seminoma foci and necrotic areas. After surgery, the AFP levels went down to normal. Investigations performed after surgery (CT scan and AFP) remained negative for the 5-year follow-up.

Seven years after the initial diagnosis, the patient started to complain of cough and chest pain. The chest radiograph revealed a mediastinal enlargement and the subsequent CT scans revealed the presence of a new 128×54×87 mm mass in the mediastinum. APF was 713 ng/mL.

A new surgery was performed, and the histologic analysis demonstrated a GCT composed of mature teratoma (90%) with multiple foci of immature teratoma with neuroectodermal differentiation and a single focus of seminoma. As no yolk sac element was recognized; the increase in AFP should be linked to the immature teratoma components.^8^ Surgical margins in the pericaval region resulted in positive for teratoma tissue. The patient started a new follow-up program and remains without evidence of disease 1 year after surgery.

When he was 16 years old, a testicular volume low for his age was detected and genetic advice was requested. The karyotype demonstrated a diagnosis of KS.

## DISCUSSION

KS has an incidence of 1 in 600 males, it is characterized by a 47XXY karyotype and an abnormal sex differentiation with hypergonadotropic hypogonadism, small testes, and infertility. In childhood, KS may be undiagnosed because the phenotypic features appear during puberty.^9^

An increased incidence of different malignancies is well documented in KS patients, especially breast cancer,^10^ leukemia, lymphomas,^11^ and GCT.

Concerning GCT there is a 50-fold increased risk over the general population. This caused discussion on the opportunity to recommend a screening program for patients with KS, but as of now, it is not suggested.^7,12,13^ The reason for this higher incidence is not known but there are different hypotheses such as the germ cell proliferation due to hypergonadotropic hypogonadism or the escape of X inactivation of the genes in the additional chromosome.^7,14^

In KS children up to 80% of eCGT is located in the mediastinum and typically affects children 10 to 14 years old, with prevalence of mixed-type-nonseminomateus histology in adolescence and teratoma in prepuberal age.^7,12,15^ It is not clear if GCT has a different outcome when occurs in patients with KS, but some reports described a poor prognosis and the risk of postsurgery relapse, even in case of favorable histology.^16,17^

In pediatric population, the mediastinal GCT represents only 2% to 4% of all pediatric GCT. The authors estimated that one-third of male patients with mediastinal GCT have KS.^15,18^ Consequently, it is suggested to investigate the presence of KS in pediatric patients with mediastinal GCT.^7,15,19^

There is only one case report in the literature that describes GCT relapse in KS. As in our case, it was a mature teratoma with a small portion of the neuroepithelium, with serum markers increasing at diagnosis. In this case, the relapse occurred 7 months after surgery and was treated with surgery and chemotherapy, with a good short-term prognosis.^16^

In this article, we describe the occurrence of a late relapse of a mediastinal GCT in a patient with KS. We may also hypothesize this is a new malignancy in a predisposed subject. In both cases, the report highlights the importance of surveillance and suggests that in patients with GCT and KS, the follow-up should be longer than the 5 years planned for non-KS patients.

In our patient, the KS was diagnosed after the end of the planned follow-up. This information should have changed our approach leading to a prolongation of the follow-up plan and possibly to an earlier detection of the second mediastinal lesion. An earlier diagnosis may have allowed a complete surgical tumor resection, unfeasible in our case due to the mass extension.

Another challenge is how to extend the surveillance without significantly affecting the patient’s life and avoiding unnecessary investigations. Recent studies demonstrated that serum tumor markers are an effective and safe relapse detection method for malignant GCT, with the ability to identify more than 95% of recurrence in patients with increased markers at diagnosis.^20^ This may be true for all secreting GCT, as it was in our case. Surveillance with serum markers and infrequent chest radiographs may allow a longer follow-up reducing the biologic risk of radiologic examinations^21^ and may be recommended in patients with cancer predisposition syndromes, such as KS.

## CONCLUSIONS

**FIGURE 1:**
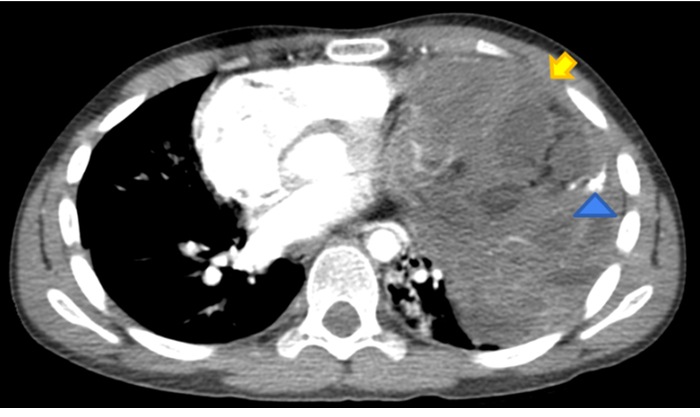
Axial contrast-enhanced chest CT performed at diagnosis showing a large (11×9×15 cm) left-side well-demarcated inhomogeneous soft-tissue density mass (*yellow arrow*) with calcification (*blue arrow*) displacing the adjacent structures. Radiologic features were consistent with teratoma.

**FIGURE 2:**
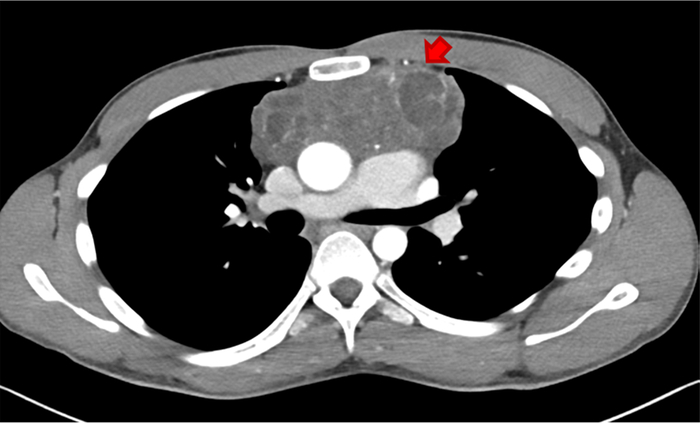
Contrast-enhanced chest CT demonstrating an inhomogeneous soft-tissue density mass (*red arrow*) located in anterior mediastinum. This finding is referable to recurrence of teratoma.

With the limitations of a single case experience, our report confirms the importance of investigating the presence of KS in males with a diagnosis of primary mediastinal GCT and suggests the need for a personalized and longer follow-up strategy in syndromic patients with GCT (Figs. 1 and 2).

## References

[1] Shaikh F Murray MJ Amatruda JF. Paediatric extracranial germ-cell tumours. Lancet Oncol. 2016;17:e149–e162. 27300675 10.1016/S1470-2045(15)00545-8

[2] Göbel U Schneider DT Calaminus G. Germ-cell tumors in childhood and adolescence. Ann Oncol. 2000;11:263–272. 10811491 10.1023/a:1008360523160

[3] Kaatsch P Häfner C Calaminus G. Pediatric germ cell tumors from 1987 to 2011: incidence rates, time trends, and survival. Pediatrics. 2015;135:e136–e143. 25489016 10.1542/peds.2014-1989

[4] Terenziani M Barretta F Gattuso G. Should we reduce routine surveillance imaging in pediatric germ cell tumors? Pediatr Blood Cancer. 2023;70. Assessed 2023 Apr 14. https://onlinelibrary.wiley.com/doi/10.1002/pbc.30200 10.1002/pbc.30200 36625403

[5] Honecker F Aparicio J Berney D. ESMO Consensus Conference on testicular germ cell cancer: diagnosis, treatment and follow-up. Ann Oncol. 2018;29:1658–1686. 30113631 10.1093/annonc/mdy217

[6] De Sanctis V Fiscina B Soliman A. Klinefelter syndrome and cancer: from childhood to adulthood. Pediatr Endocrinol Rev. 2013;11:44–50. 24079078

[7] Bonouvrie K van der Werff ten Bosch J van den Akker M. Klinefelter syndrome and germ cell tumors: review of the literature. Int J Pediatr Endocrinol. 2020;2020:18. 33005196 10.1186/s13633-020-00088-0 PMC7526209

[8] Mann JR Gray ES Thornton C. Mature and immature extracranial teratomas in children: the UK Children’s Cancer Study Group experience. JCO. 2008;26:3590–3597. 10.1200/JCO.2008.16.0622 18541896

[9] Pradhan D Kaman L Dhillon J. Mediastinal mixed germ cell tumor in an infertile male with Klinefelter syndrome: a case report and literature review. J Can Res Ther. 2015;11:1034. 10.4103/0973-1482.150697 26881632

[10] Brinton LA. Breast cancer risk among patients with Klinefelter syndrome: breast cancer among Klinefelter patients. Acta Paediatrica. 2011;100:814–818. 21241366 10.1111/j.1651-2227.2010.02131.x PMC4024394

[11] Park YT Park CH Bae MA. Angioimmunoblastic T-cell lymphoma in a patient with Klinefelter syndrome. Am J Case Rep. 2016;17:529–534. 27452959 10.12659/AJCR.897572 PMC4961065

[12] Nichols CR Heerema NA Palmer C. Klinefelter’s syndrome associated with mediastinal germ cell neoplasms. JCO. 1987;5:1290–1294. 10.1200/JCO.1987.5.8.1290 3040921

[13] Hasle H Jacobsen BB Asschenfeldt P. Mediastinal germ cell tumour associated with Klinefelter syndrome: a report of case and review of the literature. Eur J Pediatr. 1992;151:735–739. 1425792 10.1007/BF01959079

[14] Chaligné R Heard E. X-chromosome inactivation in development and cancer. FEBS Letters. 2014;588:2514–2522. 24937141 10.1016/j.febslet.2014.06.023

[15] Williams LA Pankratz N Lane J. Klinefelter syndrome in males with germ cell tumors: a report from the Children’s Oncology Group. Cancer. 2018;124:3900–3908. 30291793 10.1002/cncr.31667 PMC6241518

[16] Chen CK Chang YL Jou ST. Treatment of mediastinal immature teratoma in a child with precocious puberty and Klinefelter’s syndrome. Ann Thorac Surg. 2006;82:1906–1908. 17062277 10.1016/j.athoracsur.2006.03.077

[17] Konheim JA Israel JA Delacroix SE. Klinefelter syndrome with poor risk extragonadal germ cell tumor. Urol Case Rep. 2017;10:1–3. 27800296 10.1016/j.eucr.2016.09.006 PMC5079238

[18] De Pasquale MD Crocoli A Conte M. Mediastinal germ cell tumors in pediatric patients: a report from the Italian Association of Pediatric Hematology and Oncology. Pediatr Blood Cancer. 2016;63:808–812. 26766550 10.1002/pbc.25895

[19] Rojas AP Vo DV Mwangi L. Oncologic manifestations of Klinefelter syndrome. Hormones. 2020;19:497–504. 33000452 10.1007/s42000-020-00241-7

[20] Fonseca A Xia C Lorenzo AJ. Detection of relapse by tumor markers versus imaging in children and adolescents with nongerminomatous malignant germ cell tumors: a report from the Children’s Oncology Group. JCO. 2019;37:396–402. 10.1200/JCO.18.00790 PMC6553816 30576269

[21] Miglioretti DL Johnson E Williams A. The use of computed tomography in pediatrics and the associated radiation exposure and estimated cancer risk. JAMA Pediatr. 2013;167:700. 23754213 10.1001/jamapediatrics.2013.311 PMC3936795

